# The Potential of Cold Plasma and Electromagnetic Field as Stimulators of Natural Sweeteners Biosynthesis in *Stevia rebaudiana* Bertoni

**DOI:** 10.3390/plants11050611

**Published:** 2022-02-24

**Authors:** Augustė Judickaitė, Veronika Lyushkevich, Irina Filatova, Vida Mildažienė, Rasa Žūkienė

**Affiliations:** 1 Department of Biochemistry, Faculty of Natural Sciences, Vytautas Magnus University, Vileikos Str. 8, LT-44404 Kaunas, Lithuania; auguste.judickaite@vdu.lt (A.J.); vida.mildaziene@vdu.lt (V.M.); 2 B. I. Stepanov Institute of Physics, National Academy of Sciences of Belarus, 68 Prospekt Nezavisimosti, BY-220072 Minsk, Belarus; v.lyushkevich@ifanbel.bas-net.by (V.L.); filatova@presidium.bas-net.by (I.F.)

**Keywords:** *Stevia rebaudiana* Bertoni, cold plasma, electromagnetic field, steviol glycosides

## Abstract

Stevioside (Stev) and rebaudioside A (RebA) are the most abundant steviol glycosides (SGs) responsible for the sweetness of *Stevia rabaudiana* Bertoni. As compared to Stev, RebA has a higher sweetening potency, better taste and therefore is the most preferred component of the stevia leaf extracts. The aim of this study was to determine the effect of pre-sowing seed treatment with abiotic stressors cold plasma (CP) and electromagnetic field (EMF) on the amount and ratio of RebA and Stev in the leaves of stevia. Additionally, the effect on total phenolic content, flavonoid content and antioxidant activity was investigated. Seeds were treated 5 and 7 min with cold plasma (CP5 and CP7 groups) and 10 min with electromagnetic field (EMF10 group) six days before sowing. The germination tests in vitro demonstrated that all treatments slightly increased germination rate and percentage. HPLC analysis revealed that CP and EMF had strong stimulating effect on SGs accumulation. All treatments increased RebA concentration approximately 1.6-fold; however, the ratio of RebA/Stev decreased from 8.5 in the control to 1.9, 2.5 and 1.1 in CP5, CP7 and EMF10 groups respectively, since the concentration of Stev increased more than RebA, 7.1, 4.6 and 11.0-fold, respectively, compared to control. However, treatments had opposite effect on total phenolic content, flavonoid content, and antioxidant activity. We have demonstrated for the first time that short time pre-sowing treatment of stevia seeds with CP and EMF can be a powerful tool for the enhancement of biosynthesis of RebA and Stev, however it can have negative impact on the content of other secondary metabolites.

## 1. Introduction

*Stevia rebaudiana* Bert. (Bertoni) is a perennial shrub indigenous to Paraguay, South America, and nowadays it is cultivated abundantly in many countries as economically important source of natural low-calorie sweeteners, steviol glycosides (SGs) [1]. Stevia-based sweeteners have increased in market usage due to growing consumer demand for natural products with low or no added sugars. The global stevia market was valued at USD 650 Million in 2020 and is projected to reach USD 1.28 billion by 2028, growing at a compound annual growth (CAGR) of 8.95% [2].

**Figure 1:**
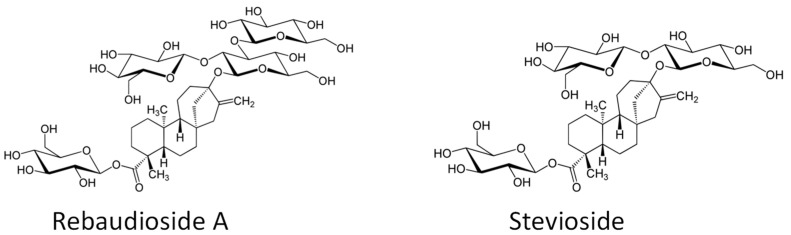
Chemical formulas of rebaudioside A and stevioside.

There are at least 38 steviol glycosides identified in stevia to date [3]. Rebaudioside A (RebA) and stevioside (Stev) are the most abundant steviol glycosides (SGs) responsible for the sweetness of stevia (Figure 1). As compared to Stev, RebA has an additional glucose monomer that gives it a higher sweetening potency and therefore RebA is the most preferred component of the stevia leaf extracts. In dried leaves, Stev and RebA account for more than 90% of the total SGs found in stevia leaves. Stev is 110–270 times sweeter than conventional sugar (sucrose) [4]. For comparison, RebA is estimated to be 140–400 times sweeter than sucrose [5]. RebA also lacks liquorice off-taste and lingering sweet aftertaste characteristic to Stev. Therefore, in stevia product industry the stimulation of RebA biosynthesis rather than Stev and higher RebA/Stev ratio is preferable. The aftertaste is eliminated if RebA and Stev are present at least in equal quantities [6].

Results of many studies and clinical trials confirmed that beside the sweet taste, stevia extract and SGs may offer a multitude of beneficial effects on health. Next to diterpenes SGs, non-sweetener fraction is rich of phenolic compounds, giving additional health benefits to the leaf material adding extra value to the product. Stevia extracts and SGs are associated with anti-hypertensive, anti-hyperglycemic, antioxidant, anti-inflammatory, antifungal, anti-microbial activities, and anti-cariogenic action (reviewed in [7]). Due to these various beneficial attributes and absence of side effects in long term use, sweeteners produced from stevia plants are gaining popularity.

Increased demand for stevia products forces the search of new methods for enhancement of economical and ecological production. Different methods and their combinations to increase SG yield are used: selection and breeding of cultivars with high concentrations of SGs, micropropagation [6], optimization of cultivation conditions (photoperiod length, moisture, temperature) [8], fertilization including biofertilizers [9], treatment with nanoparticles [10,11], and modifying after-harvest procedures such as drying, extraction conditions, and enzymatic conversion of Stev to RebA [12,13,14]. The main disadvantages of these methods often are time-consuming, expensive, or polluting, growing conditions that are climate-dependent and therefore, are not easily modified. Stevia propagation is also problematic: seed germination is poor (15–50%), and vegetative propagation results in low yields. Seed treatment with a physical stressor to obtain higher quality is regarded as clean and cheap technology compared to the use of chemicals. Seed treatment with cold plasma (CP) and electromagnetic field (EMF) is an environmentally friendly method used to improve various plant properties and stimulate synthesis of secondary metabolites [15]. Non-thermal plasma or cold plasma is a non-equilibrium gas discharge plasma, consisting of charged particles, such as ions, free electrons, and neutral particles, including gas molecules, free radicals and UV photons. In cold plasma, the particles are not in thermodynamic equilibrium: electron temperature of a plasma can be several orders of magnitude higher than the temperature of the neutral species or of the ions, which is near room temperature [16]. Radiofrequency (RF) EMF is non-ionizing radiation in which the energy and momentum are carried by alternating magnetic and electric fields, and numerous effects of exposure to EMF has been documented for plants and their seeds [17]. CP and EMF applications in agriculture and food production are gaining increasing attention in recent decades’ research. Stimulating effects of these stressors on seed germination, morphometric parameters and biomass production of various plants are well described (see recent reviews [17,18,19,20,21,22]); however, much less is known about CP and EMF-induced changes in secondary metabolite biosynthesis and underlying mechanisms. We have demonstrated the potential of CP and EMF to increase the amount of vitamin C, caffeic acid derivatives and radical scavenging capacity in purple coneflower (*Echinacea purpurea*) [23], non-psychotropic canabinoids in industrial hemp (*Cannabis sativa*) [24], isoflavones in leaves [25] and root exudates [26] of red clover (*Trifolium pratense*), different secondary metabolites in common buckwheat (*Fagopyrum esculentum*) [27]. Additionally, the germination rate or yield was increased in most of the mentioned studies.

On the other hand, although CP and EMF treatment effects were assessed for numerous plant species and on various morphometric and biochemical plant traits [17,18,19,20,21,22,23,24,25,26,27], it was never applied for treatment of *Stevia rebaudiana*.

The aim of this study was to evaluate the potential of pre-sowing seed treatment with low pressure capacitively coupled CP or RF EMF for the stimulation of main steviol glycosides (RebA and Stev) biosynthesis in stevia plants. Additionally, we have investigated the impact of these treatments on seed germination and content of other secondary metabolites—phenolic compounds, flavonoids, and the resulting effects on antioxidant activity. We have reported for the first time that short time pre-sowing treatment of stevia seeds with CP and EMF can be a powerful tool for the enhancement of biosynthesis/accumulation of RebA and Stev; however, CP and EMF treatments decreased RebA/Stev ratio, the content of phenolics, flavonoids and antioxidant activity.

## 2. Results

### 2.1. Effects on Germination In Vitro

**Figure 2:**
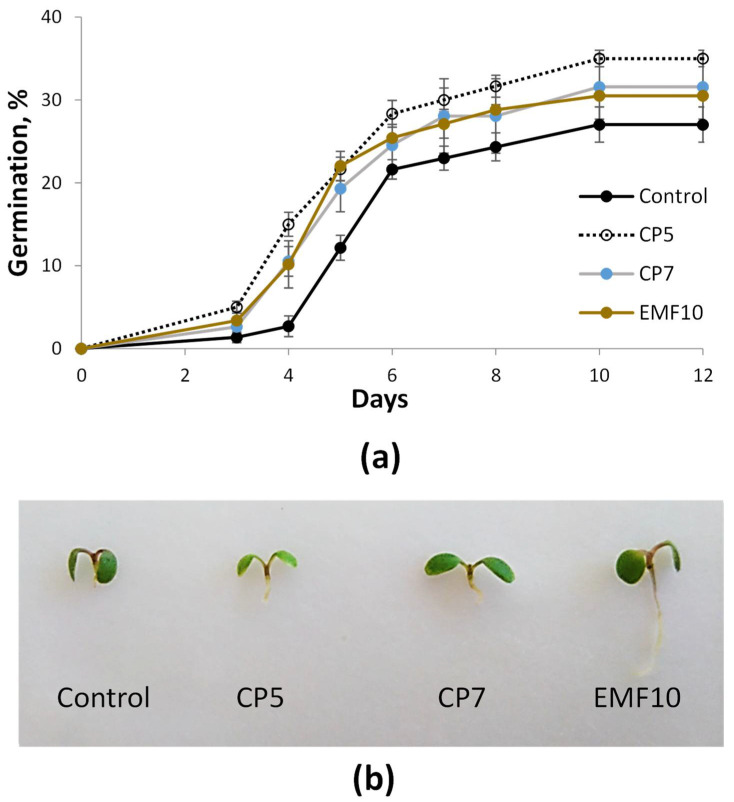
Germination kinetics of *Stevia rebaudiana* seeds (Mean ± SEM, *n* = 4) (**a**) and typical 5-day old seedlings of *Stevia rebaudiana* (**b**).

Based on some our previous studies on different plant seeds [25,26,28,29], the chosen durations for seed treatments were 5 and 7 min for cold plasma (these treatments are further abbreviated as CP5 and CP7, respectively), and 10 min for EMF treatment (this treatment is abbreviated as EMF10). The results of in vitro germination test performed 6 days after CP and EMF treatments for the control and treated seeds of *S. rebaudiana* are presented in Figure 2a. The germination curve for control group indicated slower germination rate and lower final germination percentage compared to the curves of all treated groups. Richards plots were used to determine the main indices of germination kinetics for quantitation of differences and data are presented in Table 1. All treatments decreased median germination time (Me) by ~1 day, indicating increase in the germination rate. Both cold plasma treatments (CP5 and CP7) slightly increase quartile deviation (Qu) indicating bigger dispersion of germination time (less uniform germination). The most positive response in germination percentage (Vi) was achieved in CP5 treatment group where Vi was 29% higher compared to control. CP7 and EMF treatments increased Vi 17% and 13%, respectively.

Stevia seed treatment with CP and EMF not only ameliorated germination rate and percentage but also had positive effect on seedling root development. Typical 5-day old seedlings are shown in Figure 2b. Control group had more seedlings with less developed roots (1 mm or less) compared to CP and EMF groups, and EMF group had much longer roots in comparison to all other groups.

### 2.2. Effects on Concentrations of Steviol Glycosides

**Figure 3:**
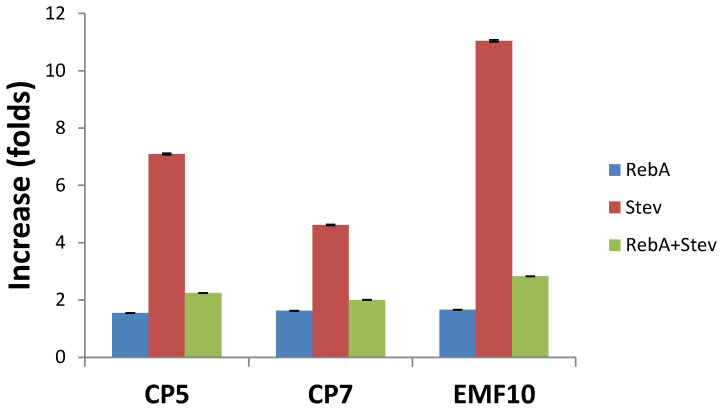
CP and EMF-induced increase in concentration of RebA, Stev and total concentration of RebA and Stev in stevia leaves compared to control. Mean ± SEM (*n* = 4), statistically significant differences compared to control (*p* ≤ 0.05) were obtained in all treatment groups.

All seed treatments considerably increased Stev and RebA concentration in leaves as compared to the control (Table 2). This increase in folds in respect to the control is shown in Figure 3. RebA concentration increased approximately 1.6-fold; however, the ratio of RebA/Stev decreased from 8.4 in the control group to 1.9, 2.5 and 1.1 in CP5, CP7 and EMF10 groups, respectively. RebA/Stev ratio inversely correlated with RebA (the linear correlation coefficient was r^2^ = 0.96), total amount of RebA and Stev (r^2^ = 0.91), but less with Stev (r^2^ = 0.77). RebA/Stev ratio was mainly affected by the concentration of Stev that increased more than RebA, 7.1, 4.6 and 11.0-fold, respectively, compared to the control (Figure 3).

### 2.3. Effects on Total Phenolic Content, Flavonoid Content, and Antioxidant Activity

**Figure 4:**
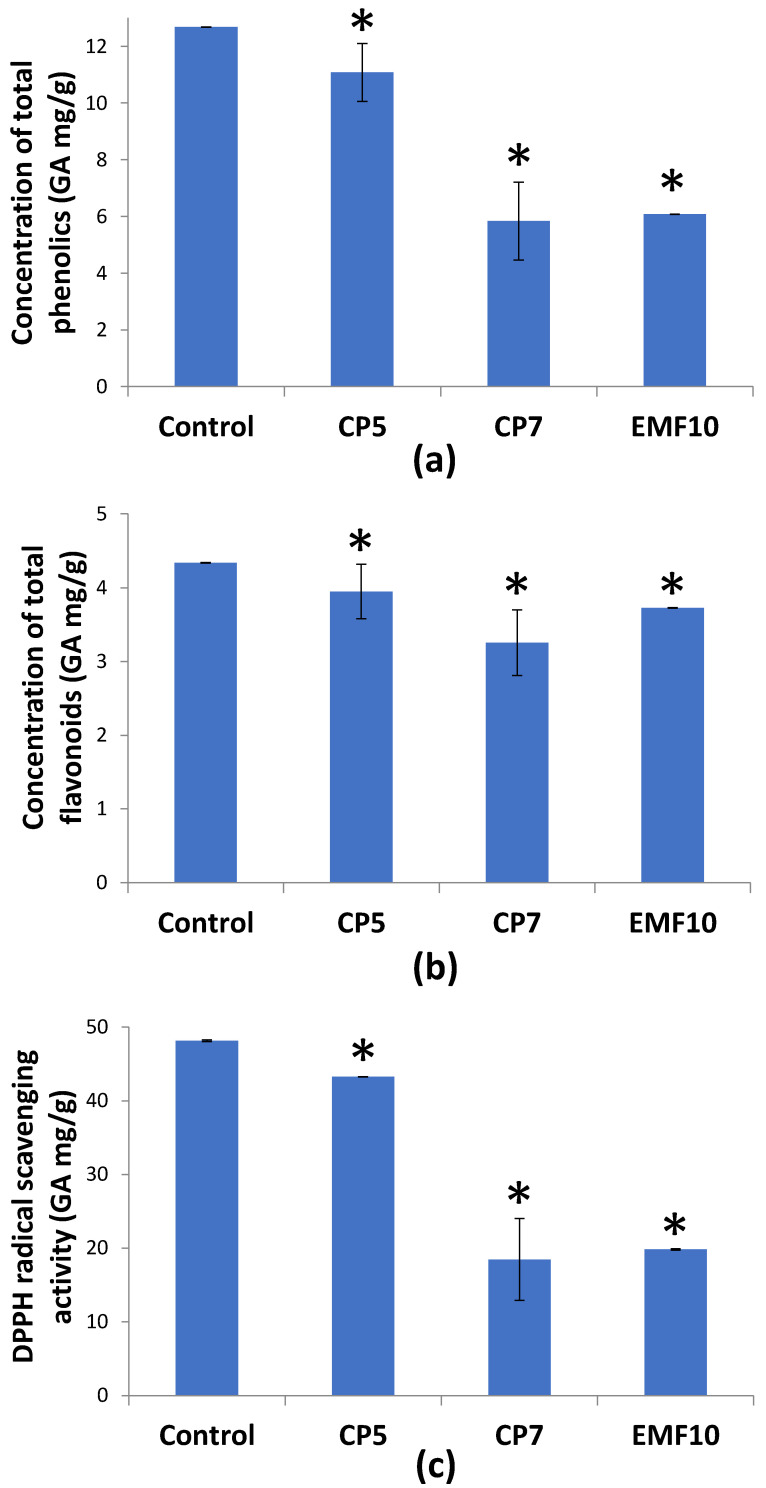
Concentration of total phenolics (**a**), flavonoids (**b**) and antioxidant activity (**c**) in stevia leaves. Mean ± SEM (*n* = 4), * statistically significant differences compared to control (*p* ≤ 0.05).

In contrast to CP and EMF-induced SGs production stimulation, these treatments had negative impact on the content of total phenolics (TPC), flavonoids (TFC) and antioxidant activity (Figure 4).

CP5 decreased TPC by 13% (Figure 4a). CP7 and EMF10 decreased the concentration much more, 2.2- and 2.1-fold, respectively. TFC was only slightly decreased by CP and EMF treatment (Figure 4b). CP7 treatment has the strongest effect (−25%).

The effect of CP and EMF treatment on antioxidant activity in stevia leaves was evaluated by measuring the scavenging of the stable 2,2-diphenyl-1-picrylhydrazyl free radical (DPPH) and the results are shown in Figure 4c. The pattern of the effect is following the pattern of changes in TPC with the correlation of r^2^ = 0.9965.

## 3. Discussion

The purpose of this study was to evaluate the potential of pre-sowing seed treatment with CP and EMF to stimulate the production of main steviol glycosides—RebA and Stev—in *Stevia rebaudiana* plant. These effects of stevia seed treatments with these physical stressors have never been studied before.

The early effect of CP or EMF manifested in stimulation of germination. Pre-sowing seed treatments with CP and EMF are known to ameliorate the germination yield and rate for seeds of numerous plant species [17,18,19,20,21,22]. Seeds of stevia are characterized by physiological dormancy but often are in a non-dormant state [30]. Hence, low germination yields of stevia seeds are not related to dormancy but are mostly caused by sterility. Therefore, a remarkable increase in germination yield was not expected in this study. Infertile achenes are formed due to sporophytic self-incompatibility. Fertile seeds have fully developed embryos and water-permeable seed coat. Stevia plants are conventionally propagated through cuttings, but this traditional method cannot produce a large number of plants and raising seedlings by sexual plant reproduction is limited [31]. Seed treatment with physical stressors increased germination yield by 13–29%. The molecular mechanism of CP- and EMF-induced germination improvement is still unclear. Some factors involved in seed response to such treatments were, however, elucidated recently. The eustress response to CP and EMF treatment is related to the increased ratio between gibberellins and abscisic acid contents [29,32]. Another well documented mechanism is CP-induced seed surface modification (mainly oxidation due to active particles present in plasma) and consequent increase in hydrophilicity and improved water uptake [33,34]. EMF does not induce such changes on seed surface, because seeds do not interact with aggressive CP-generated particles upon exposure to EMF [35].

The effects of seed treatment with two different stressors, EMF and CP, were compared in this study. CP is complex stressor consisting of the electrical component (discharge), numerous charged particles (free electrons, ions) and neutral active species, including gas molecules, free radicals, metastable particles, and generated photons (including UV) [36]. RF EMF has relatively low quantum energy and does not ionize atoms and molecules; however, EMF may activate molecules (water, components of membranes) by causing electronic excitation and increasing the frequency of collisions while penetrating through the seed tissues [17,37]. Numerous studies (reviewed in [17]) reported that effects of seed treatment with EMF can induce substantial positive effects on germination and seedling growth. Comparison of CP and EMF effects on the same plant species (including effects on the seed electron paramagnetic resonance (EPR) signal [38], seed phytohormones and protein expression in seedlings [39], content of secondary metabolites [23,24,25,26,27]) showed that, for certain plant species, EMF treatment is not less effective tool for seed priming than CP, although the reactive species are not involved in seed interaction with EMF.

Our results show that effects of seed treatment with two different stressors (CP and EMF) on the performance of stevia follow similar trends: positive effects of germination and synthesis of SGs is associated with negative effect on TPC and flavonoid amount as well as decrease in antioxidant activity. Compared to CP, EMF had stronger stimulating effects on the growth of seedling roots and induced the strongest increase in the concentration of Stev (Table 2, Figure 3). The effect of EMF on antioxidant activity and content of such antioxidants as TPC or flavonoids did not differ from the effect of CP7 and was stronger compared to effect of CP5.

Accumulation of steviol glycosides is known as a complex and dynamic process, reflecting an underlying physiological and biochemical processes that are currently intensively studied but not yet fully understood [8]. As our results show, the picture is complicated even further by the unknown action mechanisms of abiotic stressors, such as CP and EMF on the accumulation of SGs. We have demonstrated for the first time that pre-sowing seed treatment with CP and EMF can considerably increase Stev and RebA concentration in leaves (in times in the respect to the control) (Table 2) and at different extent for each SG. *Stevia rebaudiana* cultivar Criolla used in this study is a breeding product characterized by high content of RebA compared to Stev, but even in such a cultivar RebA content can be increased by a very short seed treatment. Unfortunately, the RebA /Stev ratio was not increased in this study. The result “quantity over quality” was obtained since the total SGs content increased mainly due to stronger increase in Stev compared to RebA concentration: RebA concentration increased only 1.6-fold, while the concentration of Stev increased 4.6-11.0 -fold depending on treatment (Figure 3). In this study we have determined that EMF treatment for 10 min is the most effective treatment concerning SGs production stimulation in stevia from all treatments used. Nevertheless, current data do not permit to presuppose that CP treatment is less effective and cannot be a promising method. CP5 treatment results in higher Stev and total SGs concentrations compared to CP7 (Table 2) meaning that 7-min treatment with CP can be already too long and the optimal duration to be determined could be less than 5 min and give similar results to EMF treatment. In addition, to further determine the yield of SGs in industrial applications, the dynamics of SGs concentration and consequential crop performance and adaptivity to environmental conditions must be evaluated, i.e., effects on plant growth, morphology, biomass per plant or per soil area, leaf-to-stem ratio, etc. We did not observe CP- or EMF-induced changes in plant biomass in investigated 8-week-old plants; however, the optimal harvest time is at later vegetative stages were the dynamics of the induced changes on a longer time scale should be evaluated.

SGs biosynthesis is complex and poorly understood process. Therefore, further investigations by applying combined approaches of transcriptomics, RNomics, proteomics, metabolomics and fluxomics are required to extend understanding of the mechanism of CP- and EMF-induced SGs accumulation. Lucho et al. [40] demonstrated the lack of correlation between stevioside content and the transcription of the corresponding biosynthetic genes in their study, what, according to Kumar and others [41], may also be due to the fact that the up-regulated genes (or their gene products) are nonlimiting/nonregulatory. Parallel to this, Saifi and others [42] identified two miRNAs that may up-regulate, and nine miRNAs that may down-regulate their target genes of the steviol glycosides biosynthetic pathway in *S. rebaudiana*. Overall, there is scarce information about the regulation of the SG biosynthesis pathways and master switches for this regulation.

In contrast to CP and EMF-induced SGs production stimulation, these treatments had negative impact on the content of total phenolics (TPC), flavonoids (TFC) and antioxidant activity. Various abiotic physical and chemical stressors usually simultaneously increase production of SGs and phenolic compounds. Such effects were demonstrated for PEG 6000-stimulated drought stress [43]. The large amount of secondary metabolites in *S. rebaudiana* provoke considerations about the role of SGs in plant adaptivity and overlapping of SGs functions with those of phenolic compounds, especially given the significant metabolic cost.

SGs play important role in the adaptation of plants to stress environments via alleviating stress associated effects [44,45]. Libik-Konieczny et al. [46] presented a hypothesis that steviol glycosides might function in the protection of photosynthetic apparatus against adverse environmental conditions. A standard literature states that the primary function of polyphenols is also to act as UV protection agents in plants [47,48]. However, this theory was criticized by Kuhnert and Karaköse [49], since they could not correlate the sunshine hours to phenolics nor a single flavonoid or a single chlorogenic acid, except for cis-caffeoyl derivatives. Ceunen and Geuns [8] presented several hypotheses about SGs function in a plant: steviol, the aglycone of SGs, could act as a gibberellin precursor; SGs synthesis is a defense mechanism against insects; SGs might serve as a long-term energy reserve and might play a role in the cellular antioxidant network since they have the capacity to act as potent scavengers of reactive oxygen species; however, all these hypothesis remains inconclusive.

**Figure 5:**
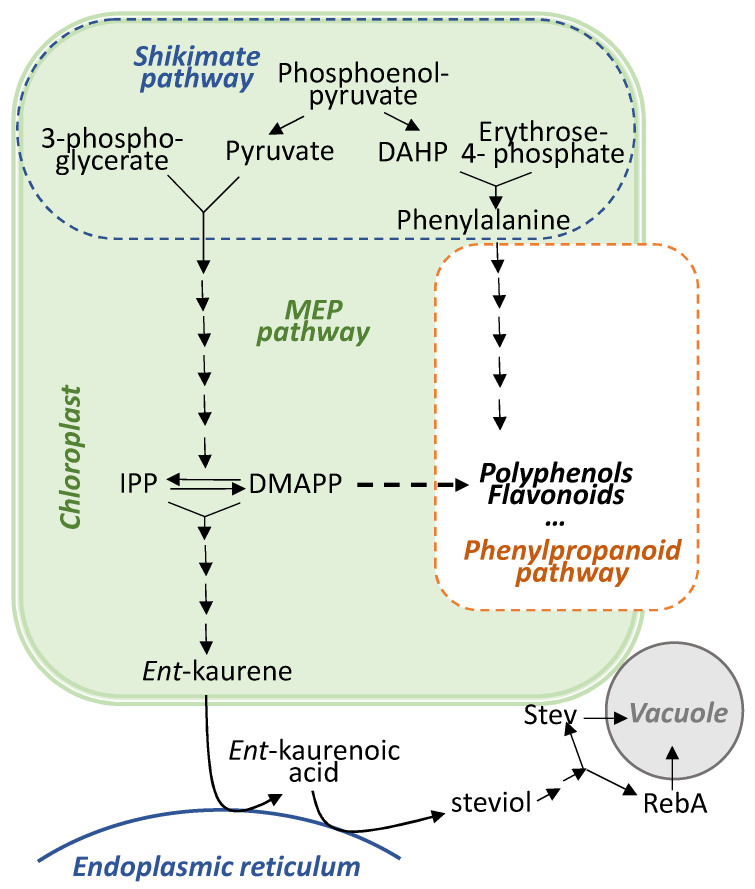
Schematic overview of terpenoid and flavonoid/ polyphenols biosynthetic pathways. The names of compounds are as follows: DAHP, 3-deoxy-D-arabino-heptulosonate 7-phosphate; DMAPP, dimethylallyl diphosphate; MEP, methyl-erythritol phosphate. Lines of arrows indicate multistep reactions, dashed arrow—putative reaction direction.

The opposing effects of stressors on SGs and TPC we demonstrated in stevia (increase in SGs content and decrease in TPC) were not observed by other authors. Nevertheless, the tendency found in this study was reproducible in similar study using other type of cold plasma (dielectric barrier discharge plasma) treatment and different varieties of stevia (unpublished data). Libik-Konieczny et al. [46], however, demonstrated the opposite reaction of stevia to adverse environmental conditions, i.e., decrease in SGs amount, but similar tendency of inversely proportional changes in SGs and TPC—the climatic stress resulted in lower SGs production but higher TPC. There is little information on the crosstalk of the SG biosynthesis pathways with biosynthetic pathways of phenolic compounds and flavonoids. Plastid-derived terpenoids SGs are synthesized by the methyl-erythritol phosphate (MEP) pathway (Figure 5), whereas phenolic compounds and flavonoids—via the shikimate/phenylpropanoid pathway. The terpenoid and flavonoid biosynthetic pathways are supplied with carbon skeletons generated in a primary metabolism but, otherwise, are thought to operate independently of one another in most plant tissues [50] (Figure 5). One of the possible explanations for the opposite effects on biosynthesis of SGs and phenolic or flavonoid compounds (as well as antioxidant activity, which is determined by the phenylpropanoids) observed in this study is competition between MEP and phenylpropanoid pathways for the common precursor phosphoenolpyruvate. The last decade’s studies, however, are beginning to uncover metabolic and regulatory connections between these two major branches of the specialized plant metabolism. Some metabolites from MEP pathway such as DMAPP can be used for the synthesis of both flavonoids and terpenoids [51,52,53,54]. Moreover, certain transcription factors coordinate metabolic activities between the flavonoid and terpenoid biosynthetic pathways [55,56]. Clearly, much more research must be done to uncover all levels of regulation of metabolic pathways in order to explain fundamentals and targets of CP and EMF action and gain knowledge for treatment protocol development and effect prediction.

Whatever molecular mechanisms are involved in the observed effects, our findings provide a new promising tool for manipulation in SGs’ production in *Stevia rebaudiana*. Future research could be directed to find out the optimal treatment dose/duration, to investigate the persistence of the effects in the time course of treated seed storage, the dynamics of the induced changes during different vegetation stages and optimal harvest time, the trait transfer possibility to vegetatively or sexually propagated plants, and the reproducibility of the effect by applying different plasma sources on different cultivars of *Stevia rebaudiana*.

## 4. Materials and Methods

### 4.1. Chemicals and Reagents

The standard of rutin, galic acid, Folin-Ciocalteu’s phenol reagent, HPLC grade methanol, 2,2-diphenyl-1-picrylhydrazyl, ethanol were obtained from Sigma Aldrich (St.Louis, MO, USA), stevioside and rebaudioside A were from TransMIT (Geiben, Germany), HPLC-grade acetonitrile, sodium acetate were from Sharlau Chemie S. A. (Sentmenat, Spain), HCl, sodium carbonate, acetic acid were from Carl Roth (Karlsruhe, Germany), hexamethylenetetramine, aluminum chloride were from Thermo Fisher Scientific (Lankashire, UK). All solutions were prepared with ultrapure 18.2 MΩ water from a Watek ultrapure water purification system (Watek Ltd., Ledeč nad Sázavou, Czech Republic).

### 4.2. Plant Material

Seeds of *Stevia rebaudiana* Bertoni cultivar Criolla were received from Academy of Agricultural Sciences, Vytautas Magnus University (Kaunas, Lithuania). Seed quality was checked using a stereomicroscope, and pale seeds were removed as sterile, thus, dark seeds only were used for experiments.

### 4.3. Seed Treatment with CP and EMF

**Figure 6:**
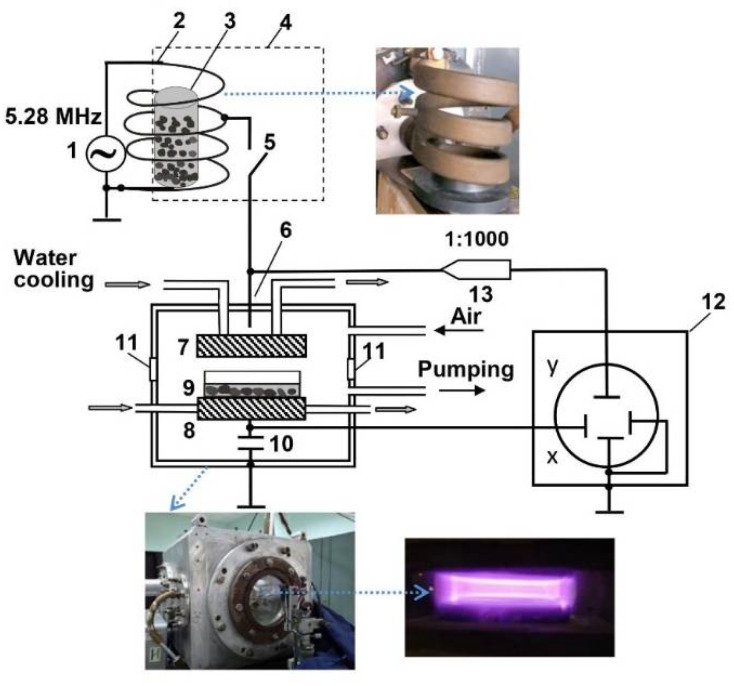
Schematic diagram of the experimental setup: 1—RF generator, 2—inductor (the view right), 3—dielectric container with seeds, 4—screen, 5—commutator, 6—vacuum chamber (the view is shown below), 7—powered electrode, 8—lower electrode, 9—Petri dish with seeds, 10—measuring capacitor, 11—window (the view of ignited plasma is shown below right), 12—oscilloscope, 13—voltage probe.

A schematic diagram of the experimental setup for seed treatment with CP and RF EMF is presented in Figure 6.

Low pressure capacitively coupled plasma was produced in a planar geometry reactor consisting of two water-cooled copper electrodes placed at 20 mm from each other in a stainless-steel hermetic chamber. RF voltage was applied to the upper electrode by the commutator 5 (Figure 6). Plasma diagnostic methods including optical emission spectroscopy (OES) based on nitrogen emissions and the discharge characteristic measurement is used to control plasma parameters during the treatments. The effective electron temperature T_e_ was determined using the technique based on the measurement of the ratio I^391^/I^394^ of the peak intensities of emission of the ionic N_2_^+^ (λ = 391.4 nm) and molecular N_2_ (λ = 394.3 nm) nitrogen bands [57]. Emission spectra were recorded in the range from 220 to 950 nm by a spectrometer SL100 (SOL Instruments Ltd.) equipped with a CCD area image sensor S10141 (Hamamatsu Photonics Norden AB, Sweden). Typical emission spectra of air plasma can be found elsewhere [24,58]. The effective electron density n_e_ was estimated from the expression: j = n_e_⋅e⋅v_d_, where j—current density, v_d_—drift velocity of the electrons, e—electron charge. The current density j was calculated as j = I/S, where I is the discharge current, S is the area of the electrode. Experimental setup for measurement the discharge characteristics is presented in [59]. Open sterile glass Petri dish with evenly dispersed 100 seeds was placed on a grounded electrode. Before igniting the discharge (parameters are given in Table 3), the air was pumped from the chamber for about 7 min to reach the working pressure.

Seed treatment with RF EMF was carried out by placing the dielectric container with seeds in three-turn water-cooled coil of the RF generator. The characteristics of EMF are shown in Table 3, the electric and magnetic strength components in the axial zone of the coil were as described previously [35]. The treatment was performed in ambient air at atmospheric pressure and room temperature. Based on some our previous studies on different plant seeds [25,26,28,29], the chosen duration for seed treatments was 5 and 7 min for cold plasma (this treatment is further abbreviated as CP5 and CP7, respectively), and 10 min for EMF treatment (this treatment is abbreviated as EMF10).

Control, CP- and EMF-treated seeds were stored at room temperature (19–22 °C) for 6 days until sowing in vitro.

### 4.4. Seed Germination Test

The untreated (control) seeds and seeds exposed to EMF and CP were evenly distributed on two layers of filter paper in 90-mm-diameter plastic Petri dishes (four replicates of 25 seeds each) and watered with 5 mL distilled water. Petri dishes with seeds were placed in a climatic chamber (Pol-Eko-Aparatura KK 750, Poland) with automatic control of relative humidity (60%), light, and temperature. Alternating light regimes were maintained in the chamber (16 h light, 8 h dark) and constant temperature of 25 ± 1 °C. Seeds were provided additional water, if necessary, to prevent drying. A seed was considered germinated when the initial emergence of the radicle was observed. Germinated seeds were counted daily until their number stopped increasing.

The effects of stressors on germination were estimated by the induced changes in parameters of germination kinetics, derived using application of Richards’ function [60] for the analysis of germinating seed population [61]: Vi (%)— the final germination percentage indicating seed viability, Me (days)—the median germination time (t_50%_) indicating the germination halftime of a seed lot or germination rate, and Qu (days)—the quartile deviation indicating the dispersion of germination time in a seed lot (half of seeds with an average growth time germinate in the range Me ± Qu).

### 4.5. Plant Cultivation

After germination, seedlings were carefully transferred from Petri dishes to plastic growth containers filled with substrate BioSoil (SIA “Green-PIK LAT”, reg. No. K0.02-1386-16, Latvia), consisting of high-quality vermicompost, moss peat and sand. Characteristics: nitrogen (N)—0.3%, phosphorus (P_2_O_5_)—0.2%, potassium (K_2_O)—0.3%, organic substances—min 14.6%, humidity—max 30%, pH 6–7. Seedlings were planted in 9 × 9 × 10 cm containers and grown under greenhouse conditions, with long day photoperiod (16 h light, 8 h dark), relative humidity of 60% and constant temperature of 25 ± 1 °C.

### 4.6. Extract Preparation

The leaves were collected from 8-week-old plants and dried at 40 °C for 24 h. Dried leaves were powdered using a batch mill with disposable grinding chamber (Tube-Mill control, IKA, Staufen, Germany), and 1 g of powder was mixed with 50 mL of 70% ethanol. The extraction was carried out in triplicate by sonication for 60 min at 25 °C. The mixture was centrifuged at 16,000× *g* for 10 min, the supernatant was collected and kept at −20 °C until analysis.

### 4.7. HPLC Analysis of Steviol Glycosides

Steviol glycosides rebaudioside A (RebA) and stevioside (Stev) were separated and quantified using high-performance liquid chromatography (HPLC) [62]. An Agilent 1200 series HPLC system (Agilent Technologies Inc., Santa Clara, CA, USA) with a diode array detector was used. Samples were filtered through a syringe filter with a PVDF membrane (pore diameter 0.22 µm) and separated on a reversed phase column (Purospher STAR RP-18e 5 µm Hibar 2 × 250 mm, Merck, Germany) with a precolumn. Injection volume was 10 µL at 70 °C column temperature. Isocratic elution at a flow rate of 0.25 mL min^−1^ with a mobile phase consisting of 70% deionized water acidified with HCl to pH 2.75 and 30% acetonitrile was used for separation with an additional washing step with 50% acetonitrile. RebA and Stev were detected at the wavelength of 210 nm. Calibration was done by plotting the peak area responses against the concentration values in the concentration range from 1 to 1000 µg mL^−1^ with linear dependence for both analytes. Each analysis was repeated three times, and the mean value was used.

### 4.8. Determination of Total Phenolic Content

Total phenolic content was determined using the modified Folin–Ciocalteu method [62]. An amount of 0.2 mL of stevia extract was mixed with 1 mL of 0.2 N Folin–Ciocalteu reagent and 0.8 mL 7.5% sodium carbonate solution. After 60 min of incubation in the dark at room temperature, absorbance was measured at 760 nm. Gallic acid was used as a standard, and results were expressed by mg of gallic acid equivalent (GAE) mg g^−1^ of dry weight (DW).

### 4.9. Determination of Total Flavonoid Content

Total flavonoid content was analyzed by a colorimetric method based on the complexation of phenolic compounds with Al (III) [62]. An amount of 80 µL of stevia extract was mixed with 1920 µL of a reagent containing 40% ethanol, 0.7% acetic acid, 0.4% hexamethylenetetramine, and 0.6% aluminum chloride. After 30 min of incubation in the dark at 4 °C, absorbance was measured at 407 nm. Rutin was used as a standard, and results were expressed by mg of rutin equivalent (RUE) mg g^−1^ of DW.

### 4.10. Determination of Antioxidant Activity

Antioxidant activity was measured based on the scavenging of the stable 2,2-diphenyl-1-picrylhydrazyl free radical (DPPH) as described [62]. Accordingly, 50 µL of stevia extract were mixed with 1950 µL of a DPPH solution (0.025 mg mL, prepared in acetonitrile:methanol:sodium acetate buffer (100 mM, pH 5.5) (1:1:2)). After 15 min of incubation in the dark at room temperature, absorbance was measured at 515 nm. Rutin was used as a standard, and antioxidant activity was expressed by mg of rutin equivalent (RUE) mg g^−1^ of DW.

### 4.11. Statistical Analysis

Statistical analysis of the results was performed using Statistica 10 software (issued by IBM Lietuva, Vilnius, Lithuania to Vytautas Magnus University). Data are presented as means ± SEM (*n* = 4). The number of measured plants in the control and treatment groups varied from 24 to 33. Statistical significance of CP and EMF effects was evaluated using Student’s t-test (unpaired). The differences were assumed to be statistically significant when *p* < 0.05.

## 5. Patents

Patent application LT2020 560 “The method for the increase of steviol glycosides amount in stevia plants by seed treatment with cold plasma before sowing” (3 December 2020) at The State Patent Bureau of the Republic of Lithuania resulted from the work reported in this manuscript (inventors: Rasa Žūkienė, Vida Mildažienė).

## Figures and Tables

**Table 1:** Effect of CP and EMF on *Stevia rebaudiana* germination kinetics indices.

Group	Vi, %	Me, Days	Qu, Days
Control	27.03 ± 2.13	5.06 ± 0.12	0.68 ± 0.10
CP5	35.00 ± 1.01 *	4.39 ± 0.18 *	1.14 ± 0.08 *
CP7	31.58 ± 3.90 *	4.59 ± 0.09 *	0.98 ± 0.17 *
EMF10	30.51 ± 0.26 *	4.36 ± 0.19 *	0.79 ± 0.09

Vi, the final germination percentage; Me, the median germination time; Qu, the quartile deviation; Mean ± SEM (*n* = 4); * statistically significant difference compared to control (*p* < 0.05).

**Table 2:** Effect of CP and EMF on *Stevia rebaudiana* leaf steviol glycoside content (mg·g^−1^ of DW) and ratio (Mean ± SEM, *n* = 4).

	RebA	Stev	RebA+Stev	RebA/Stev	RebA/(RebA+Stev)	Stev/(RebA+Stev)
Control	36.71 ± 3.10	5.27 ± 1.63	41.98 ± 4.71	8.35 ± 1.62	0.88 ± 0.02	0.12 ± 0.02
CP5	56.63 ± 9.07 *	37.35 ± 8.83 *	93.99 ± 17.89 *	1.86 ± 0.24 *	0.64 ± 0.03 *	0.36 ± 0.03 *
CP7	59.58 ± 9.12 *	24.35 ± 4.14 *	83.93 ± 13.25 *	2.50 ± 0.07 *	0.71 ± 0.01 *	0.29 ± 0.01 *
EMF10	60.77 ± 0.33 *	58.15 ± 0.15 *	118.93 ± 0.18 *	1.05 ± 0.01 *	0.51 ± 0.00 *	0.49 ± 0.00 *

* statistically significant difference compared to control (*p* < 0.05).

**Table 3:** Values of different parameters of CP and EMF applied in *Stevia rebaudiana* seed treatment.

Parameter	Value
*CP treatment*	
Discharge frequency	5.28 MHz
Pressure	200 Pa
Input power	~8.4W
Effective electron temperature (T_e_)	~2.3 eV
Effective electron density (n_e_)	~5 × 10^8^ cm^−3^
Electrode diameter	120 mm
Distance between electrodes	20 mm
*EMF treatment*	
Frequency	5.28 MHz
Pressure	Atmospheric
Amplitude value of the magnetic component	835 A/m (*B* ≈ 1 mT)
Amplitude value of the electric component	17.96 kV/m

## Data Availability

Not applicable.
